# Role and modulation of perioperative stress response to improve outcomes

**DOI:** 10.1186/s40635-026-00907-3

**Published:** 2026-05-08

**Authors:** Aarne Feldheiser, Nandor Marczin

**Affiliations:** 1 Department of Anaesthesiology, Intensive Care Medicine, and Pain Therapy, Evang. Kliniken Essen-Mitte, Huyssens-Stiftung/Knappschaft, Henricistraße 92, 45136 Essen, Germany; 2 Department of Anaesthesiology, Intensive Care Medicine, and Pain Therapy, University Hospital Knappschaftskrankenhaus Bochum, Bochum, Germany; 3 Division of Anaesthesia, Pain Medicine and Intensive Care, Imperial College London, London, UK; 4 Department of Anaesthesia and Critical Care, Harefield Hospital, Royal Brompton & Harefield Hospitals, Guy’s & St. Thomas’ NHS Foundation Trust, London, UK; 5 Department of Anesthesia and Intensive Care, Semmelweis University, Budapest, Hungary

Surgery is a central pillar of care in every hospital around the world. However, postoperative complications and mortality remain unacceptably high especially after high-risk surgeries even in high-income settings [1]. Available global data suggests that at least 4.2 million people die within 30 days after surgery each year, and half of these deaths occur in high-income settings. This number makes it the third-greatest contributor to deaths after ischaemic heart disease and stroke [2]. In contrast to myocardial infarctions and stroke, patients generally consent for undergoing surgery. At the hospital level, surgeries play additionally a critical role in financial health, as surgical services are a significant contributor to hospital income, being responsible for 40–70% of hospital revenues [3].

Physiologically, surgery triggers a local and systemic host response that has evolved to limit injury and aid recovery following the surgical insult. In the field of sepsis this pattern is well characterised as the host response to various pathogens. However, adaptation of this physiological concept to surgery should also consider non-infectious pathways to sterile tissue trauma. In the end, it is a complex physiological reaction to surgical trauma, characterised by neuroendocrine, metabolic, endothelial, and immunological affections. An exaggerated local and systemic stress response serves as the principal theory of postoperative complications manifested as either single organ and multiorgan dysfunction contributing to adverse outcomes, morbidity and mortality. Owing to its complex nature, it requires a multi-modal approach to reduce the stress response and cannot be targeted by a single golden bullet.

Considering the different pathophysiological consequences, the hypothalamic–pituitary-adrenal axis primarily mediates the neuroendocrine response, leading to increased secretion of adrenocorticotropic hormone and cortisol. This hormonal surge is accompanied by sympathetic nervous system activation, resulting in elevated catecholamine levels. All these activations can cause or contribute to organ dysfunction, leading to complications [4, 5].

The metabolic response showed that insulin resistance due to perioperative stress develops as a graded response related to the magnitude of the operation. It lasts for weeks, even after medium-size surgery, and affects protein and fat metabolism [6]. An analysis showed that insulin resistance is an independent predictor of length of stay. Along with the type of surgery and blood loss during surgery, this parameter explains more than 70% of the variation in length of hospital stay [6].

The perioperative stress response also involves a significant inflammatory component, with activation of various immune cells and the release of a spectrum of pro-inflammatory mediators including a plethora of cytokines such as IL-6 and TNF-α, which further stimulate the HPA axis and contribute to systemic inflammation and immune modulation [7, 8]. Many of these activators also serve as modulators of the cellular and tissue redox status by promoting oxidative and nitrosative stress and altering endogenous antioxidant capacity and protective mechanisms.

Despite such accepted general understanding, the concept of exaggerated stress response and uncontrolled systemic inflammatory response theory remains at crossroads. There remains controversy about the magnitude of this response to invasive surgeries. The underlying mechanisms of susceptibility or ability to withstand these responses on an individual basis remain largely unknown. Most research has focused on individual components of the stress response rather than global understanding of the balance of injuries and protective pathways. Clinical trials targeting these individual components and global strategies both yielded disappointing results and currently there are no routine pharmacological and therapeutic tools to ameliorate uncontrolled stress and the systemic inflammatory response syndrome.

This collection is designed to address these major gaps and to provide a publication platform for studies to broaden our global knowledge and horizon on the pathophysiological features of perioperative stress response and on our better understanding of its clinical role and best strategies to modulate adverse events and to improve perioperative and long-term outcomes.

The goal of any perioperative care program must be a reduction of postoperative complications avoiding personal sufferings of the patients, reducing the workload of the care teams, and leading to a healthy financial situation. Much recent activities have centred on the so-called enhanced recovery after surgery (ERAS) programs as they have reduced complications, shortened hospital length of stay, and improved long-term mortality [6]. These positive effects were based on a question asked by Prof. Dr Hendrik Kehlet: why is the patient still in hospital [9]. His analysis of the pathophysiological findings of the perioperative stress response led to the interdisciplinary, interprofessional, multi-modal approach of the ERAS programs to reduce or prevent their consequences.

However, even two decades later, postoperative morbidity and mortality remain part of the business of surgical programs. A key to further improve our perioperative care should therefore be to describe the pathophysiological regulation and perhaps better understand the involved pathways as targets for future therapies. Such delineation should take into consideration the impact of the entire patient journey representing the preoperative period, the surgical phase and postoperative stage and events triggered by anaesthesia, surgery and intensive care management. As these events span different disciplines (e.g. surgery, anaesthesiology, intensive care medicine) and involve multiple professions (e.g. physicians, nurses, nutritionist) we invite submissions by any members of the perioperative community.

We are particularly seeking contributions challenging and significantly advancing the field of perioperative stress response. The submission may particularly target candidate pathways or focus on more global phenotypes, biomarkers and diagnostic and new therapeutic potential. We shall welcome original articles focussing on either preclinical models or describing human studies with observational or clinical trial design. We will consider in depth review articles, but case reports will unlikely meet the goals of this topical collection.

## Data Availability

Not applicable for this manuscript.
